# Study of nasal mucosa histopathological changes in patients with hypersensitivity pneumonitis

**DOI:** 10.1038/s41598-023-35871-5

**Published:** 2023-05-31

**Authors:** Yosri Akl, Eman Kamal Ibrahim, Tareq Muhammad Algarf, Rasha R. Mostafa, Hoda M. Abdel-Hamid, Asmaa Ibrahim Muhammed

**Affiliations:** Kasr Alainy, Faculty of Medicine, Cairo University, Almaadi, Cairo, Egypt

**Keywords:** Diseases, Medical research, Signs and symptoms

## Abstract

Hypersensitivity pneumonitis (HP) is an interstitial lung disease that develops after inhalation of a variety antigens in susceptible individuals. The nasal mucosa is constantly exposed to these antigens that can irritate the respiratory mucosa. So, the purpose of this study was to study nasal histopathological changes in order to identify any shared pathological changes between the upper airways and the well-known pathological features of HP. 40 HP patients diagnosed at the Chest Department, Kasr Alainy hospital following ATS/JRS/ALAT guidelines were included. Patients were subjected to thorough history, high-resolution computed tomography, spirometry, cough evaluation test (CET), sinonasal outcome test-22 (SNOT-22), sinonasal examination and nasal mucosal biopsy by an otolaryngologist under visualization by a rigid nasal endoscope. The mean age of the patients was 46.2 ± 13.5 (85% were females and 15% were males). 90% of patients presented with cough and the mean CET was 17.15 ± 5.59.77.5% of patients suffered from sinonasal symptoms and the mean SNOT-22 was 12.18 ± 3.8. There was a significant correlation between the burden of sinonasal symptoms represented by the SNOT-22 and the severity of the cough represented by CET (r 0.40, p 0.01). 87.5% of HP patients had chronic inflammation of the nasal mucosa with predominant lymphocytic infiltration in 72.5% of patients. 77.5% of HP patients had a high burden of sinonasal symptoms which is positively associated with cough severity. 72.5% of patients had predominately lymphocytic infiltration of the nasal mucosa.

Trial registration: retrospectively registered, registration number is NCT05723796, date of registration 13/02/2023.

## Introduction

Hypersensitivity pneumonitis is an interstitial lung disease developed from an immune-mediated reaction in predisposed and sensitized individuals to a large variety of inhaled organic and inorganic antigens^1^. The nasal mucosa is constantly subjected to these inhaled antigens and depending on the extent of inhaled antigens and their physical as well as chemical properties, these agents can cause inflammation and sensitization of the respiratory mucosa^2^.

Initial observations of the coexistence of allergic rhinitis, chronic rhinosinusitis, and asthma led to the formulation of the concept of united airways diseases (UAD)^3^. Both the upper and lower airways share macroscopic and microscopic pathological features, with a similar allergic response in rhinitis and asthma^4^.

Furthermore, there is increasing evidence that the UAD concept also applies to other chronic respiratory airway diseases, such as COPD, bronchiectasis, and cystic fibrosis^5^. Besides, it could be a presenting manifestation of granulomatous diseases such as Wegener’s granulomatosis, Churg–Strauss syndrome, or sarcoidosis^6^. Since the upper airway mucosa is the most accessible portion of the respiratory tract, it is a valuable source of respiratory tissue that could be used in the diagnosis of such cases^7^.

In both acute and chronic rhinosinusitis, the most severe and prevalent symptoms are nasal blockage, facial pain, anosmia, ageusia, and postnasal discharge. In addition, fatigue and arousing up exhausted are prevalent and worrisome^8^.

Rhinosinusitis as well has impacted the quality of life in a significant percentage of HP patients as it aggravates their cough. Treatment of cough, whenever possible, is targeted at the treatment of the underlying interstitial lung disease in addition to targeting co-factors such as rhinosinusitis^9^.

As sinonasal symptoms add to the burden of respiratory diseases and impair patients' quality of life as well as that the main purpose of the diagnosis of ILD is to make an assured diagnosis using the least invasive approach. So, the purpose of this study was to assess the burden of sinonasal symptoms in HP patients and to study nasal histopathological changes in order to identify any shared pathological changes between the upper airways and the well-known pathological features of HP.

## Methods

### Study design and participants

A cross-sectional study was conducted on 40 patients suffering from hypersensitivity pneumonitis. Patients were recruited from the Chest Department at Kasr Alainy Hospital, Cairo University during the period between 1/July 2021 to 31/October 2022. Cairo University had approved the study proposal (code: MD-138-2021).

Diagnosis of Hypersensitivity Pneumonitis was implemented based on ATS/JRS/ALAT clinical practice guidelines that require an integration of several domains in the context of a multidisciplinary discussion^10^. The defining criteria for definite, high-confidence, moderate-confidence, and low-confidence HP diagnoses are as follows; definite in patients with an identified exposure, typical high-resolution computed tomography (HRCT) pattern, BAL (bronchoalveolar lavage) lymphocytosis, and histopathological features of HP; high-confidence in patients with an identified exposure, typical HRCT pattern, and BAL lymphocytosis; moderate-confidence and low-confidence diagnoses in patients with any other combination of exposure history, HRCT pattern, and BAL analysis.

### Inclusion criteria

This study was eligible for any adult patient (> 18 years old) with definite or high-confidence diagnosis of HP.

### Exclusion criteria

Those with other interstitial lung diseases (ILD) than HP and patients with moderate or low confidence HP diagnosis as well as patients who refused to participate or had acute respiratory failure or bleeding tendency.

**Figure 1:**
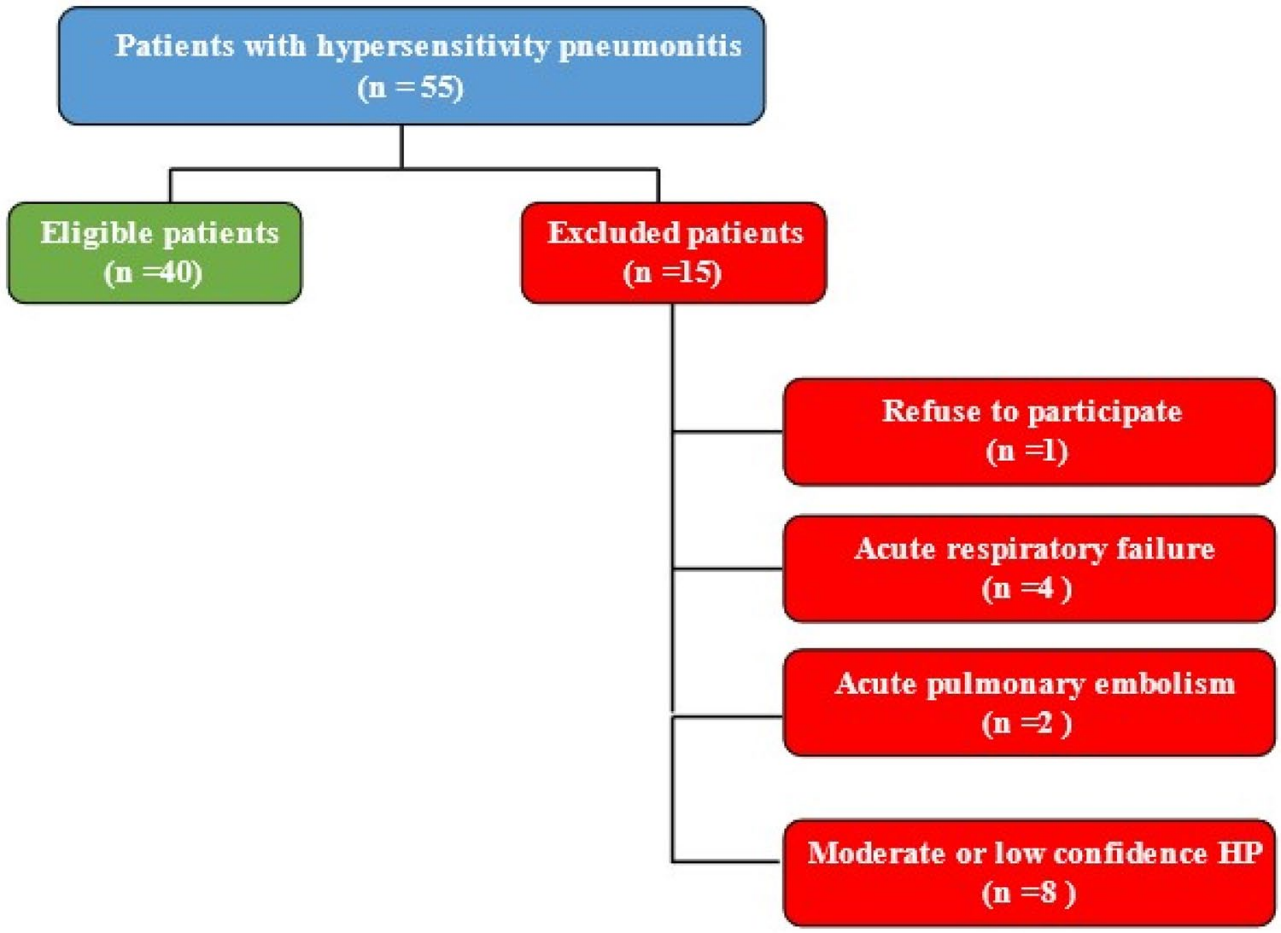
Flow diagram for the eligible and excluded patients.

Out of 55 patients with hypersensitivity pneumonitis, 15 were excluded (1 refused to participate, four had acute respiratory failure and 2 had an acute pulmonary embolism and were on anticoagulation, eight had moderate or low confidence HP) (Fig. 1).

### Data collection

We collected data on patient demographics, smoking status, and environmental and occupational exposures. In addition to baseline clinical data, an expert pulmonologist and otorhinologist made a detailed descriptive analysis of presenting upper and lower respiratory symptoms through face-to-face interviews. The cough evaluation test (CET) was used to evaluate the impact of chronic cough on physical, psychological, and social aspects^11^. Other causes of chronic cough were excluded such as gastroesophageal reflux, chronic bronchitis and drug induced cough. The modified Medical Research Council (mMRC) scale was used to grade the severity of dyspnea^12^. The SNOT-22 includes 22 items on nasal and non-nasal symptoms and is the most commonly established patient-reported outcome measure of sinonasal illness, HP patients are then classified based on the burden of sinonasal symptoms by the SNOT-22. A score of ≥ 11 was defined as a high burden of sinonasal symptoms while a score < 11 was defined as a low burden^13^.

Spirometry and 6 min walk test (6 MWT) were performed to evaluate pulmonary function.

Fiberoptic bronchoscopy with BAL cellular analysis with/without TBLB (transbronchial lung biopsy) together with HRCT were performed to establish the diagnosis of HP.

An expert otorhinologist performed nasal examination with nasal endoscopy and obtained a nasal biopsy under visualization by a rigid nasal endoscope from the level of the inferior turbinate using Blakesleytru-cut forceps to detect sinonasal affection in those patients and to evaluate the undergoing pathology, whether it's consistent with the histopathology of HP or irrelevant. An expert pathologist examined the biopsies for the presence of granulomas, the existence of underlying inflammation, and the type of predominant inflammatory cell.

### Statistical analysis

Data were registered using the statistical package for the Social Sciences (SPSS) and then were summed up using mean and standard deviation for quantitative variables, frequencies, and relative frequencies for categorical variables. An unpaired *t* test was used in normally distributed quantitative variables to compare patients with a high and low burden of sinonasal symptoms while a non-parametric Mann–Whitney test was used for non-normally distributed quantitative variables. The Chi-square (χ^2^) test was accomplished when comparing categorical data. Correlations between quantitative variables have been carried out using the spearman correlation coefficient. P-values less than 0.05 were considered statistically relevant.

### Ethics approval and consent to participate

The research ethics committee of the Chest department, at Cairo University, approved the study proposal (code: MD-138-2021). Before each patient was enrolled, the purpose and nature of the study were discussed and all participants provided informed written consent. The policy on data confidentiality was strictly adhered to. The design of the study complied with the biomedical ethics criteria of the Revised Helsinki Declaration.

## Results

The current study was conducted on 40 patients who were diagnosed with HP, the mean age of patients was 46.2 ± 13.5 years. The study included 6 (15%) males and 34 (85%) females. 80% of patients had history of exposure to birds. The main presenting pulmonary symptoms were dyspnea, cyanosis, dry cough, and productive cough (100%, 50%, 67.5%, and 22.5% respectively) with mean CET = 17.15 ± 5.59. Regarding nasal symptoms it was found that 70% of patients had a blocked nose, 7.5% had a runny nose and 5% had sneezing with mean SNOT-22 = 12.18 ± 3.8. Other clinical characteristics of the study group were summarized in Table 1.

**Table 1:** Clinical characteristics, Nasal endoscopy, and Pathological findings of nasal biopsy of the study population.

Study population (n = 40)	
Age [mean (SD)]	46.2 ± 13.5
Gender [n (%)]	
Males	6 (15%)
Females	34 (85%)
Smokers [n (%)]	0 (0%)
HP classification [n (%)]	
Non-fibrotic	10 (25%)
Fibrotic	30 (75%)
Duration of symptoms (years) [median (IQR)]	3 (2–4)
Presence of clubbing [n (%)]	29 (72.5%)
Presenting pulmonary symptoms [n (%)]	
Dyspnea	40 (100%)
Cyanosis	20 (50%)
Dry cough	27 (67.5%)
Productive cough	9 (22.5%)
CET [mean (SD)]	17.15 ± 5.59
Nasal symptoms [n (%)]	
Blocked nose	28 (70%)
Runny nose	3 (7.5%)
Sneezing	2 (5%)
Decreased sense of smell or taste	0 (0%)
Thick nasal discharge or debris	0 (0%)
SNOT-22 [mean (SD)]	12.18 ± 3.8
6MWT [mean (SD)]	
6 min walk distance (m)	180 (± 102)
Oxygen saturation on room air	91 (± 4.17)
Oxygen saturation post-exertion	81.8 (± 7.09)
Oxygen desaturation [n (%)]	36 (90%)
Spirometry [mean (SD)]	
FEV1%	53.7 (± 18)
FVC %	50 (± 16.7)
FEV1/FVC %	88 (± 10.6)
MMEF 25–75%	55.3 (± 24.7)
Nasal endoscopy and pathological findings of nasal biopsy [n (%)]	
Positive nasal endoscopy of congestion	14 (35%)
Presence of granuloma in nasal biopsy	0 (0%)
Chronic inflammation in nasal biopsy	35 (87.5%)
Predominant inflammatory cell	
Lymphocytes	29 (72.5%)
Plasma cells	0 (0%)
Eosinophils	5 (12.5%)
Neutrophils	1 (2.5%)

*SD* standard deviation, *IQR* interquartile range, *CET* cough evaluation test, *SNOT* sinonasal outcome test.

**Figure 2:**
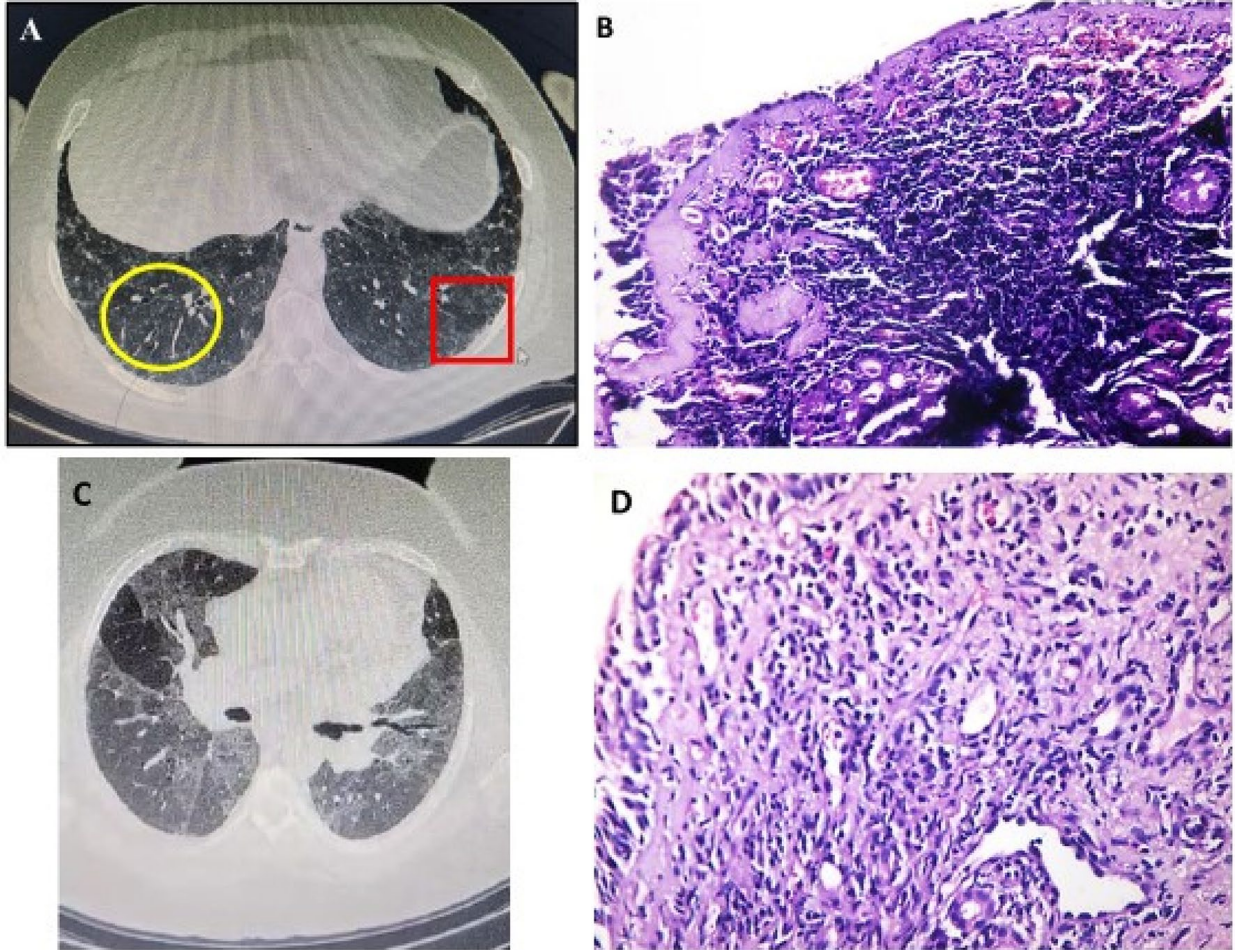
HRCT chest of a 45-year-old female patient, with a history of raising birds, presented with gradual progressive dyspnea and dry cough for 1 year. (**A**) HRCT shows a pattern of non-fibrotic HP in the form of diffuse bilateral centrilobular nodules (yellow circle) and air trapping (red square). (**B**) Nasal biopsy showed dense lymphocytic infiltration. H&E stain, original magnification × 200. CT chest of a 50 years old female patient presented with dyspnea and dry cough for 5 years. (**C**) HRCT shows a pattern of fibrotic HP in the form of bilateral mosaicism, areas of bronchiectasis, and reticulations. (**D**) Nasal biopsy showed dense lymphocytic infiltration. H&E stain, original magnification × 200.

Examination by nasal endoscopy revealed mucosal congestion in 35% of patients, However, by histopathological examination, chronic inflammation was noticed in 87.5% of patients, and lymphocytes were the predominant inflammatory cells in 72.5% of patients (Table 1, Fig. 2).

HP patients are then classified based on the burden of sinonasal symptoms by the SNOT-22. A score of ≥ 11 was defined as a high burden of sinonasal symptoms (n = 31 patients) while a score < 11 was defined as a low burden (n = 9 patients). HP patients with a high sinonasal burden were significantly older with a mean age of 48.81 ± 12.82 (*p*-value = 0.022). 61.3% of HP patients with a high sinonasal burden experienced cyanosis compared to HP patients with a low sinonasal burden with a p-value = 0.02. Other clinical characteristics of the study groups were summarized in Table 2.

**Table 2:** Clinical characteristics, Nasal endoscopy, and Pathological findings of nasal biopsy of HP Patients by the burden of sinonasal symptoms.

	HP with a high burden of sinonasal symptoms n = 31	HP with a low burden of sinonasal symptoms n = 9	P-value
Age [mean (SD)]	48.81 ± 12.82	37.22 ± 12.78	0.02
Gender [n (%)]			
Males	4 (12.9%)	2 (22.2%)	0.6
Females	27 (87.1%)	7 (77.8%)	
HP classification			
Non-fibrotic	6 (19.4%)	4 (44.4%)	0.19
Fibrotic	25 (80.6%)	5 (55.6%)	
Duration of symptoms (years) [median (IQR)]	3 (2–4)	2 (2–3)	0.086
Presence of clubbing [n (%)]	22 (71%)	7 (77.8%)	1
Presenting pulmonary symptoms [n (%)]			
Dyspnea	31 (100%)	9 (100%)	N/A
Cyanosis	19 (61.3%)	1 (11.1%)	**0.02**
Dry cough	23 (74.2%)	4 (44.4%)	0.1
Productive cough	7 (22.6%)	2 (22.2%)	1
CET [mean (SD)]	18 ± 4.6	14 ± 7.6	0.17
6MWT [mean (SD)]			
6 min walk distance (m)	181.6 ± 105.74	175 ± 96.9	0.849
Oxygen saturation on room air	91.1 ± 3.9	92.4 ± 4.9	0.41
Oxygen saturation post-exertion	81.7 ± 6.4	82 ± 9.4	0.934
Oxygen desaturation [n (%)]	28 (90.3%)	8 (88.9%)	1
Spirometry [mean (SD)]			
FEV1%	52.6 ± 15.47	57 ± 25.7	0.56
FVC %	50.03 ± 15.75	50.22 ± 20.85	0.97
FEV1/FVC %	88.23 ± 10.92	87.33 ± 9.96	0.82
MMEF 25–75%	53.58 ± 23.38	61.11 ± 29.56	0.54
Nasal endoscopy and pathological findings of nasal biopsy [n (%)]			
Positive nasal endoscopy of congestion	11 (35.5%)	3 (33.3%)	1
Presence of granuloma	0 (0%)	0 (0%)	N/A
Chronic inflammation in nasal biopsy	27 (87.1%)	8 (88.9%)	1
Predominant inflammatory cell			
Lymphocytes	23 (74.2%)	6 (66.7%)	N/A
Plasma cells	0 (0%)	0 (0%)	
Eosinophils	3 (9.7%)	2 (22.2%)	
Neutrophils	1 (3.2%)	0 (0%)	

*SD* standard deviation, *IQR* interquartile range, *CET* cough evaluation test**,** *6MWT* 6-min walk test, *FEV1* forced expiratory volume in 1 s, *FVC* forced vital capacity**,** *MMEF* maximal mid-expiratory flow, P-value significant if < 0.05.

No significant difference was found between both groups regarding nasal endoscopy findings and histopathological examination of the nasal mucosa (Table 2).

**Figure 3:**
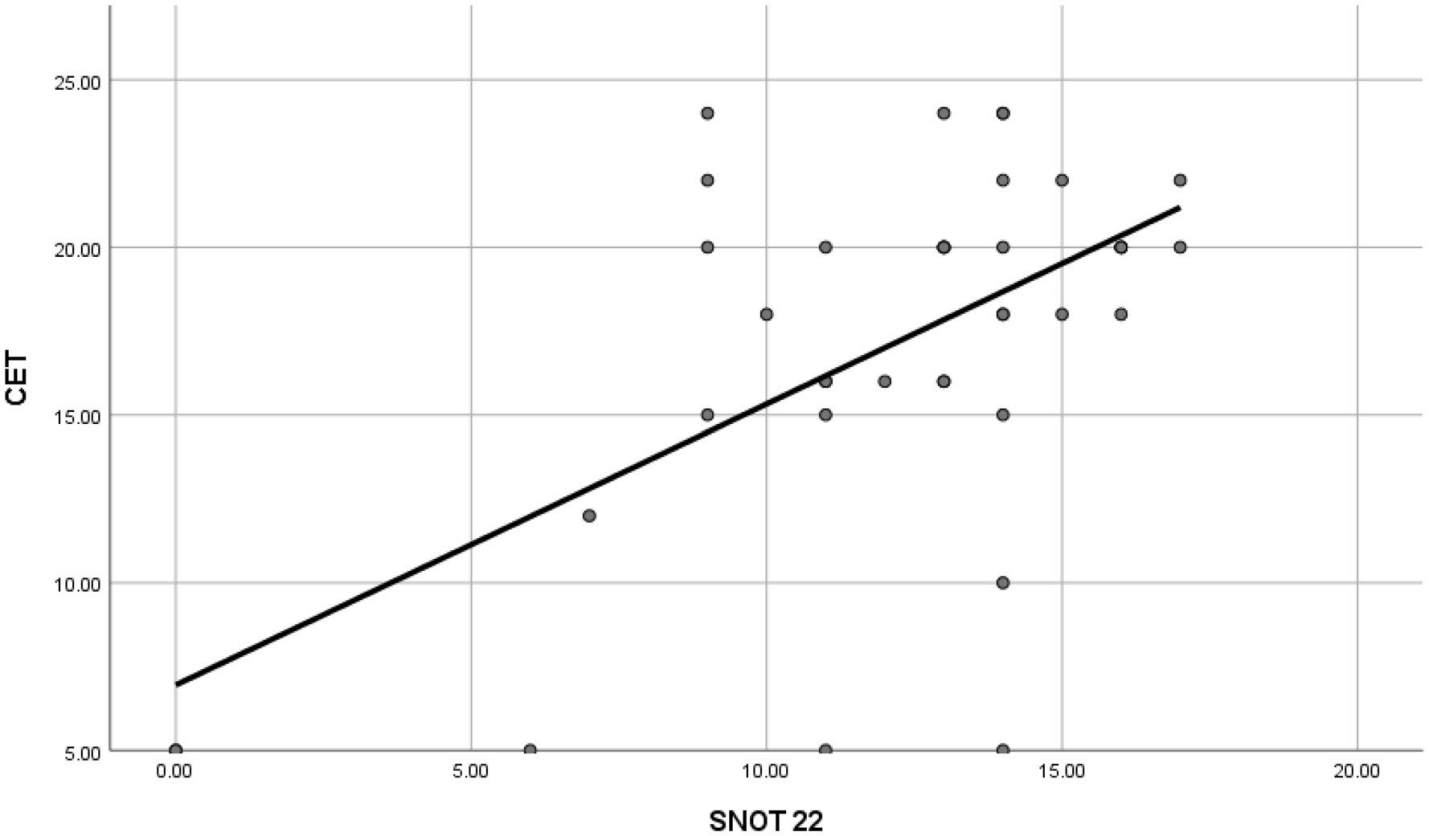
Correlation between sinonasal outcome test-22 (SNOT-22) and cough evaluation test (CET) in the study population.

There was a significant correlation between the burden of sinonasal symptoms as assessed by the SNOT-22 and the severity of cough as assessed by the CET (*r *0.4, *p*-value = 0.01) (Table 3; Fig. 3).

**Table 3:** Correlation between the burden of sinonasal symptoms and functional assessment parameters of HP patients.

	mMRC	CET	6 min walk distance (m)	FEV1%	FVC %	FEV1/FVC %	MMEF 25–75%
SNOT-22							
Correlation coefficient (*r*)	− 0.110	0.401	0.081	− 0.125	− 0.072	0.113	0.028
P-value	0.499	0.010	0.619	0.442	0.659	0.486	0.864

*mMRC* modified Medical Research Council, *CET* cough evaluation test, *6MWD* 6-min walk distance, *FEV1* forced expiratory volume in 1 s, *FVC* forced vital capacity**,** *MMEF* maximal mid-expiratory flow, *SNOT* sino-nasal-outcome-test, P-value significant if < 0.05.

## Discussion

Hypersensitivity pneumonitis (HP) is an interstitial lung disease that develops after inhalation of organic or inorganic antigens in susceptible individuals^1^. The nasal mucosa is constantly exposed to these antigens that can irritate the respiratory mucosa^2^. To our knowledge, it is the first study to assess the burden of sinonasal symptoms in patients with HP and to study histopathological changes of the nasal mucosa and its relation to the well-established pathological changes of HP.

The mean age of patients was 46.2 ± 13.5 years. The study included 6 (15%) males and 34 (85%) females. This is consistent with previous Egyptian studies which reported female predominance in HP^14,15^.

Identifying the aetiology of hypersensitivity pneumonitis is difficult due to the wide variability of different antigens. Bird-related HP is likely to be the most frequent subtype of HP^1^. In our study, 80% of patients had identified exposure which was bird raising, this was in line with Said et al. and Viktor et al.^16,17^. In contrast, other studies did not find an identified exposure in 60–70% of their patients^14,18^.

Chronic cough has a major impact on the quality of life in patients with ILD, including HP patients**.** Many conditions could increase the intensity of cough in ILD including rhinosinusitis and GERD (gastroesophageal reflux disease). Treatment of the underlying interstitial process, rhinosinusitis, and gastro-oesophageal reflux are the main targets that can lead to improvement of cough and hence improve the patient’s quality of life^9^.

The present study revealed that 67.5% of patients suffering from dry cough, 22.5% from productive cough with mean CET = 17.15 ± 5.5, and 70% of patients had blocked noses, with mean SNOT-22 = 12.18 ± 3.8 (Supplementary Table 1).

Lymphocytosis is a characteristic finding in HP patients. Morisset and co-workers reported consensus among international experts for high-confidence HP diagnosis without performing lung biopsy in the right clinical contest (recognized exposure and HRCT consistent with HP) when BAL lymphocytosis was > 40%^19^.

It’s important to notice that despite the normal appearance of the nasal mucosa by endoscopy, chronic congestion, and inflammation were noticed by histopathological examination in 87.5% of patients. Regardless of the absence of granuloma and epithelioid cells in all patients, it’s important to mark that the predominant inflammatory cells in 72.5% of patients were lymphocytes.

It is well known that bronchoalveolar lavage (BAL) lymphocytosis and histopathologic data are an integral part of the diagnosis of HP which requires multidisciplinary discussion based on clinical and radiologic findings as well^10^.

Previous data on asthmatic patients revealed that inflammation of nasal mucosa is characterized by eosinophilic inflammation and local IgE production, hence the formulation of the concept of united airways diseases (UAD)in asthmatic patients^20^. Moreover, there is increasing evidence that the UAD concept also applies to other chronic respiratory tract diseases, such as COPD, bronchiectasis, and cystic fibrosis^5^.

Authors classify the study population based on the burden of sinonasal symptoms by SNOT-22 into two groups; HP with a high burden of sinonasal symptoms (SNOT-22 ≥ 11) included 31 patients and HP with a low burden of sinonasal symptoms (SNOT-22 < 11) included 9 patients. HP patients with ahigh sinonasal burden were significantly older and more obese, and 61.3% of them experienced cyanosis in comparison to 11% only of HP with a low sinonasal burden. We did not find any significant difference between both groups regarding nasal endoscopy findings and histopathological examination of the nasal mucosa.

Our study revealed a significant correlation between the burden of sinonasal symptoms as assessed by the SNOT-22 and the severity of cough as assessed by the CET. To our knowledge, no other studies had evaluated the burden of sinonasal symptoms in HP patients, nor correlated this burden with the severity of cough and dyspnea in those patients.

Based on this finding we suggest that proper treatment of sinonasal symptoms may have a favourable effect on controlling cough severity in those patient, however further cohort study is needed to confirm this assumption.

The correlation between the sinonasal symptoms and the severity of respiratory symptoms was evaluated in COPD patients, and it was found that a high burden of sinonasal symptoms is positively associated with the clinical markers of symptom severity (cough, and dyspnea)^3^.

Previous studies conducted on asthmatic patients found that the severity of sinonasal symptoms was significantly correlated to the severity of asthmatic symptoms suggesting that rhinitis, sinusitis, and asthma are all manifestations of a single disease^21,22^.

The strength of our study is that it is the first study to assess the burden of sinonasal symptoms in patients with HP and to study histopathological changes of the nasal mucosa and its relation to the well-established pathological changes of HP.

This study had several limitations. (1) It is a single centre study. (2) Small number of the study group. (3) We did not evaluate the direct relation between the causative antigen and nasal symptoms and/or pathology. (4) We did not study the effect of antigen avoidance and steroid treatment (systemic/intra-nasal) on nasal symptoms.

## Conclusion

It was concluded that 77.5% of HP patients included in the study had a high burden of sinonasal symptoms, there was a significant correlation between the burden of sinonasal symptoms represented by the SNOT-22 and the severity of the cough represented by CET. 72.5% of HP patients had predominately lymphocytic infiltration of the nasal mucosa.

## Supplementary Information

Supplementary Table 1.

## Data Availability

The datasets used and/or analyzed during the current study are available from the corresponding author upon reasonable request.

## Abbreviations

HP: Hypersensitivity pneumonitis
BAL: Bronchoalveolar lavage
ATS/JRS/ALAT: The American Thoracic Society/Japanese Respiratory Society/Latin American Thoracic Association
SNOT-22: Sinonasal outcome test-22
CET: Cough evaluation test
UAD: United airways diseases
COPD: Chronic obstructive pulmonary disease
ILD: Interstitial lung disease
HRCT: High-resolution computed tomography
mMRC: The modified Medical Research Council
6 MWT: 6 Min walk test
TBLB: Transbronchial lung biopsy
SPSS: The statistical package for the social sciences
GERD: Gastroesophageal reflux disease
