# Supplementary material for: Study of nasal mucosa histopathological changes in patients with hypersensitivity pneumonitis
Source: Sci Rep. 2023 May 31;13:8868. doi: 10.1038/s41598-023-35871-5 (PMC10232410; doi:10.1038/s41598-023-35871-5)
Supplement: Supplementary file 1 — Supplementary Table 1. [file 41598_2023_35871_MOESM1_ESM.docx]

|  | **Fibrotic**  **n= 30** | **Non-fibrotic**  **n= 10** | **P-value** |
| --- | --- | --- | --- |
| **Presenting pulmonary Symptoms [n (%)]** | | | |
| **Dyspnea** | 30 (100%) | 10 (100%) | --- |
| **Dry cough** | 22 (73.3%) | 5 (50%) | 0.246 |
| **Productive cough** | 7 (23.3%) | 2 (20%) | 1 |

Supplementary table (1) Comparison of fibrotic and non-fibrotic HP regarding presenting symptoms
